# Genetic analysis of African lions (*Panthera leo*) in Zambia support movement across anthropogenic and geographical barriers

**DOI:** 10.1371/journal.pone.0217179

**Published:** 2019-05-31

**Authors:** Caitlin J. Curry, Paula A. White, James N. Derr

**Affiliations:** 1 Interdisciplinary Program of Genetics, Department of Veterinary Pathobiology, College of Veterinary Medicine and Biomedical Sciences, Texas A&M University, College Station, Texas, United States of America; 2 Center for Tropical Research, Institute of the Environment and Sustainability, University of California, Los Angeles, Los Angeles, California, United States of America; Senckenberg am Meer Deutsches Zentrum fur Marine Biodiversitatsforschung, GERMANY

## Abstract

The Luangwa Valley in eastern Zambia is a transverse offshoot of the Great Rift Valley system. This region appears to have an isolating effect as evidenced by suspected endemic subspecies, such as the Cookson’s wildebeest and Thornicroft’s giraffe. Recent mitochondrial DNA studies demonstrated that African lions in Zambia consist of two highly diverse eastern and western sub-populations. Herein, we report nuclear and mitochondrial DNA results from 409 lions that support this population substructure across Zambia but proposes only partial isolation of the Luangwa Valley with more movement between the populations than previously thought. Population assignment analysis identifies two populations with little evidence of admixture assigning lions to either the eastern or western sub-populations. A high occurrence of private alleles and clear evidence for a Wahlund effect further justify the presence of a highly structured population. But, while mitochondrial DNA analysis still shows little to no matrilineal gene flow (F_ST_ = 0.53) between sub-populations, microsatellite analysis suggests there is gene flow (F_ST_ = 0.04) with low but significant isolation-by-distance and an average of 6 migrants per generation. Evidence of isolation-by-distance is also found in factorial correspondence analysis with the Lower Zambezi National Park and eastern corridor clusters overlapping isolated clusters of the Luangwa Valley and western sub-population. From this evidence, the Luangwa Valley appears separated from the western sub-population with some dispersal through the southern regions of the eastern sub-population. Both the eastern and western sub-populations have high heterozygosity (0.68 and 0.69, respectively) and genetic diversity (0.47 and 0.50, respectively) values, indicative of genetically healthy populations.

## Introduction

Zambia has one of the largest wild lion populations with a current estimate of around 1,200 individuals [1] within a range of more than 200,000 km^2^ [2]. While lions do exist in small numbers in outlying areas of Zambia (e.g., Liuwa Plains National Park, Mweru Wantipa National Park), lion distribution can generally be divided into two regions (eastern and western) that are separated by geographic and anthropogenic features.

Zambia is one of nine countries with over 1,000 lions and has 2 of the 10 lion strongholds [3]. Kafue National Park (NP) and the adjoining Game Management Areas (GMAs) in the western part of the country collectively form the Greater Kafue Ecosystem (GKE). The GKE is designated a potential stronghold due to heavy poaching of the prey base [3], a significant concern as a decrease in prey lowers carnivore carrying capacity [4].

The Luangwa Valley Ecosystem (LVE) is a lion stronghold [3]. It is an offshoot of the Great Rift Valley system along the Luangwa River consisting of three NPs, the North Luangwa NP, South Luangwa NP, and Luambe NP, and their surrounding GMAs. The presence of suspected endemic subspecies has been used as evidence of the Luangwa Valley’s geographic isolation. This includes Thornicroft’s giraffe (*Giraffa camelopardalis thornicrofti*) found to be genetically distinct by mtDNA analyses [5]. However, more recent genetic studies using nuclear loci no longer consider them as their own subspecies and, although geographically separated by 500-km, now group them with a neighboring population of the Masai giraffe (*Giraffa camelopardalis tippelskirchi*) subspecies [6]. Other presumed endemic subspecies include the Cookson’s wildebeest (*Connochaetes taurinus cooksoni*) [7], a subspecies of Blue wildebeest and the Crawshay’s zebra (*Equus quagga crawshayi*), which has a striping pattern unique to Zambia [8].

Zambia is one of a handful of countries that actively engages in wild lion monitoring, conservation, and management [9]. Lions are a protected species under the Zambia Wildlife Act No. 12 of 1998 making it illegal to hunt, kill, capture or be in possession of a lion specimen without a permit [9]. Trophy hunting of lion is legally conducted in many of the GMAs. However, in Zambia as in many other countries, human-lion conflict is the greatest threat to lions outside of protected areas (PAs) [9]. Increased human activity on the edges of NPs and into GMAs severely limits the movement of carnivores [10,11]. Therefore, it is relevant to consider population connectivity when formulating lion conservation strategies.

**Fig 1:**
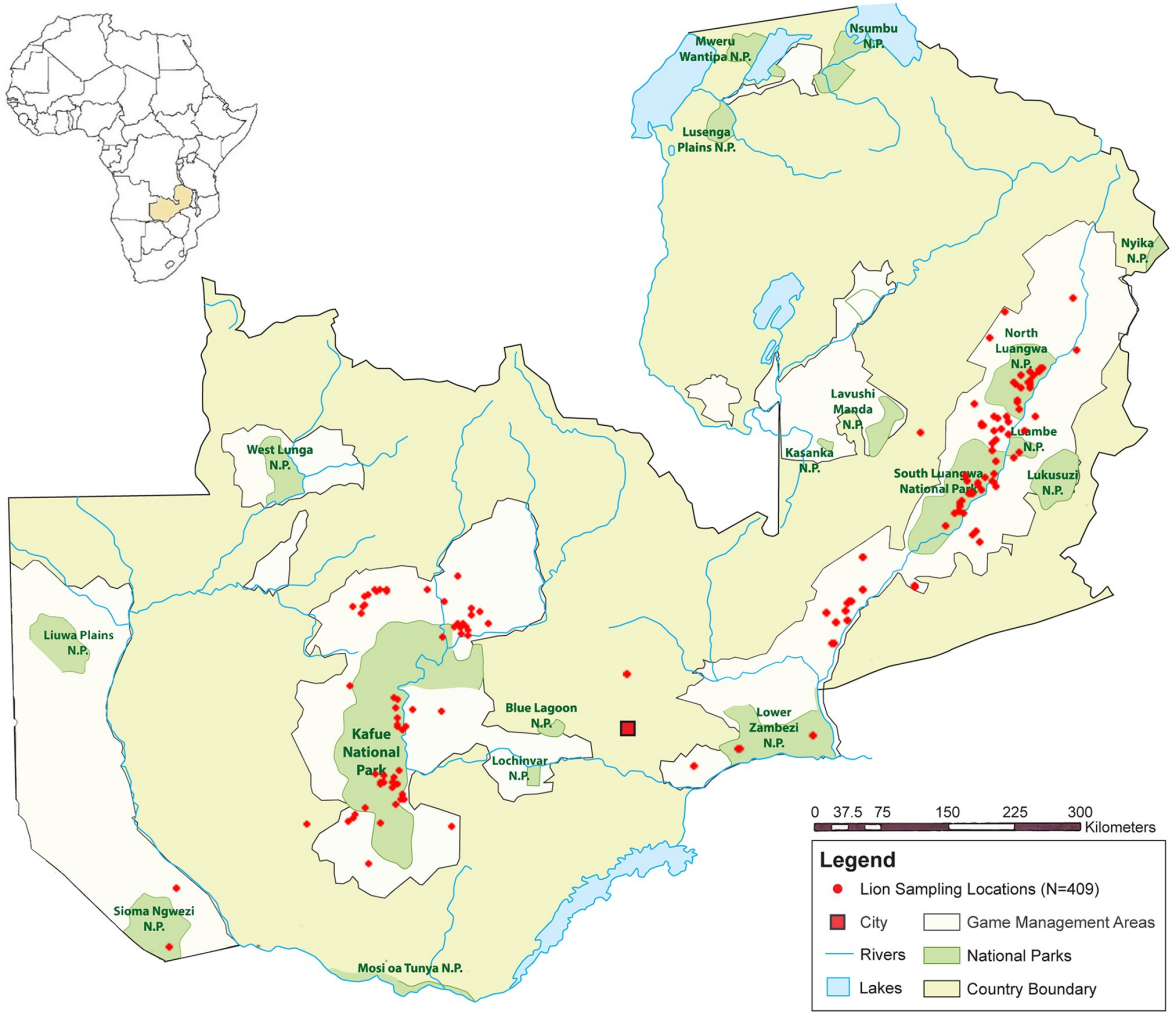
Map of Zambia with sampling locations. Sample locations are latitude and longitude coordinates of GPS location at sampling (181 lions at 181 locations) or a central location based on sampling area (228 lions at 33 locations). Detailed location information is in S1 Appendix.

Much of the area between the LVE and the GKE is comprised of an anthropogenic patchwork of towns and farms and is, therefore, considered uninhabitable by lions [3]. This is supported by mitochondrial DNA (mtDNA) which shows little to no matrilineal gene flow between the eastern and western sub-populations [12]. However, studies using minimal sampling from Zambia found an admixture pattern suggestive of male-mediated gene flow [13]. A larger study including both nuclear and mtDNA data was needed to capture a more accurate representation of lion movement and diversity in the region. This study used nuclear and mitochondrial markers from 409 lions across Zambia (Fig 1) to assess population structure and the potential for movement of lions between the Luangwa Valley and the Greater Kafue ecosystems.

## Materials and methods

### Sample collection

The Zambia Lion Project (ZLP) provided 446 samples for analysis. Samples are in the form of hair, skin, bone and/or tissue and were collected during research conducted by ZLP in partnership with the Zambia Wildlife Authority (Research/Employment Permit No. #008872). For more details on sample collection, refer to Curry *et al*. [12].

Samples were collected between 2004–2012 in five of Zambia’s National Parks (North Luangwa, South Luangwa, Lower Zambezi, Kafue and Sioma Ngwezi) and more than forty GMAs, making this dataset representative of Zambia’s countrywide lion population. Each sample has an accompanying latitude and longitude sampling location. When exact sampling location could not be provided, a central latitude/longitude was given based on the sampling locale (see S1 Appendix).

### DNA extraction

DNA was successfully extracted from 421 samples of which 409 were further analyzed for mtDNA and microsatellites. Of the 12 samples eliminated, ten were duplicate individuals and two were not lions. The 409 samples analyzed consist of 324 males, 83 females, and 2 unknown (S1 Appendix), including all individuals previously studied in Curry *et al*. [12].

DNA was extracted using standard protocols and procedures used in the DNA Technologies Core Laboratory at Texas A&M University in College Station, TX (http://vetmed.tamu.edu/dnacore). PCR amplification was done using the KAPA Biosystems KAPA2G Robust HotStart PCR Kit with protocols described in Curry *et al*. [12] for mtDNA and *Curry & Derr* [14] for microsatellites. Cycling profiles for mtDNA and microsatellite amplification can be found in S2 Appendix. PCR product was sequenced and/or genotyped on an Applied Biosystems 3130xl or 3730 Genetic Analyzer.

### Mitochondrial DNA and sequencing

Samples were amplified for a continuous region from the mtDNA 12S-16S genes using primers designed by Antunes *et al*.[15]. These primers were designed to prevent amplification of a 12.5 kb integration of mtDNA into the nuclear genome, or numt [16]. Sequences were manually edited and assigned a haplotype using SEQUENCHER v4.8 [17].

Diversity calculations and phylogenetic analyses were carried out as in Curry *et al*. [12] so a direct comparison could be made with the larger sample size. The data was analyzed using Arlequin v3.5 [18] as a full Zambian population and separated into eastern and western sub-populations. Number of polymorphic sites, gene diversity, nucleotide diversity and haplotype diversity were calculated for each. The sub-populations dataset was used for intra-population calculations of the coefficient of differentiation (F_ST_) and hierarchical analyses of molecular variance (AMOVA). Finally, pairwise differences were calculated between sampling locations.

Phylogenetic analysis included all haplotypes from Antunes *et al*. [15] (GENBANK Accession #s FJ151641-FJ151652) and Zambian haplotypes found in Curry *et al*. [12] (GENBANK Accession #s KT164799-KT164803) and this study (ENA Accession #: LR593884). Phylogenetic analysis was performed using maximum likelihood analysis in PhyML 3.0 [19] and Bayesian inference methods with Mr. Bayes [20,21]. For Bayesian analysis, samples were drawn every 1,000 steps over 50,000,000 MCMC steps with the first 10% discarded as burn-in and the tree was rooted with the tiger (*Panthera tigris*; GENBANK Accession #KJ508413) that was aligned and trimmed to the lion sequences. A haplotype network was also made using the median-joining option of Network v4.6.1.3 [22].

### Microsatellites and genotyping

A panel of 14 miniSTRs [14] (Leo006, Leo008, Leo031, Leo045, Leo077, Leo085, Leo098, Leo126, Leo224, Leo230, Leo247, Leo281, Leo391, and Leo506) were used for microsatellite analysis. Each sample was amplified, genotyped, and scored a minimum of two times at each locus to determine a consensus genotype. Samples were scored using STRand [23]. Samples scored at 10 or more loci were retained for further analysis.

Molecular diversity indices of expected and observed heterozygosity, deviation from Hardy-Weinberg equilibrium (HWE), conventional F-statistics, percentages of molecular variation, and number of migrants were calculated using Arlequin [18], GenAlEx [24], and GenePop [25]. Isolation-by-Distance (IBD) was calculated for females, males, and all individuals using the Mantel Test in GenAlEx [24]. Factorial Correspondence Analysis (FCA) and Principle Coordinate Analysis (PCoA) were carried out in GeneTix [26] and GenAlEx [24], respectively.

Number of alleles, number of private alleles and distribution of alleles were determined using GenAlEx [24] and allelic richness and private allelic richness was calculated using rarefaction in HPRare [27,28].

Effective Population Size was calculated using NeEstimator [29] using the linkage disequilibrium (LD) model. The two sub-populations have a high number of private alleles, many of which occur in low frequencies (<0.01). Because low allele frequencies can bias calculations of effective population size [30], the P_*crit*_ was set to frequencies of 0.00, 0.01, and to exclude only singleton alleles.

STRUCTURE [31] was used to evaluate population structure. Fifteen runs were performed for K = 1 to 6 using 1,000,000 MCMC reps after 100,000 burn-in. Structure Harvester [32] was used to implement the Evanno method [33] to determine ΔK. CLUMPP [34] was then used to combine replicate runs and results were visualized using Distruct [35].

## Results

A total of 409 individuals were analyzed with 398 genotype panels and 391 sequences produced. Eighteen of the 398 genotyped individuals did not produce a haplotype. This is likely due to sample degradation. Degraded samples may not allow for amplification of large amplicons [36], such as this 1800-bp sequence of 12S-16S. Eleven individuals produced a haplotype but were not genotyped because DNA was lost in an accident after sequencing. These individuals didn’t have more sample available for re-extraction of DNA.

### Mitochondrial diversity

A total of 391 samples produced full sequences (Table 1 and S1 Appendix). Distribution of haplotypes is shown in Table 1. A novel haplotype, Z6, was found in three individuals in the eastern sub-population. Even with the addition of another haplotype [12], these results support the findings of Curry *et al*. Haplotype Z6 differs from haplotype H11 by one single nucleotide polymorphism (SNP) at position 1524 (Table 2).

**Table 1:** Distribution of haplotypes. Number of males (♂), females (♀), and with unknown gender (?) for each haplotype is indicated for all areas sampled in Zambia along with the haplotype frequencies. Haplotypes H1, H9 and H11 were previously described by Antunes et al. [15]. Haplotypes Z1-Z6 are novel.

	Eastern			Western		Out				
Haplotype	♀	♂	?	♀	♂	♀	♂	n	ƒ	s.d.
H1				0	1			1	0.003	0.003
H9	0	1	0	24	81	1	1	108	0.274	0.023
H11	15	59	1	0	2	0	1	78	0.202	0.020
Z1	23	125	1	0	3	1	0	153	0.391	0.025
Z2	0	1	0					1	0.003	0.003
Z3				10	27			37	0.095	0.015
Z4				0	4			4	0.010	0.005
Z5				5	1			6	0.015	0.006
Z6	0	3	0					3	0.008	0.004
Total	38	189	2	39	119	2	2	391		
	229			158		4				
A	5			7		3		9		

"Out" indicates samples collected outside PAs between sub-populations. n = sample size; for ♂, ♀ and ? for each area and by area, haplotype and total. A = number of haplotypes. ƒ = frequency. s.d. = standard deviation

**Table 2:** Nucleotide position for each polymorphic site. All nucleotide polymorphisms in 12S-16S are shown for haplotype H1. For all other haplotypes, only nucleotides that differ from H1 are shown. Haplotypes found in Zambia are in bold.

	*242*	*369*	*393*	*462*	*513*	*531*	*571*	*596*	*631*	*684*	*695*	*732*	*798*	*812*	*840*	*841*	*928*	*929*	*961*	*1039*	*1082*	*1140*	*1220*	*1247*	*1328*	*1387*	*1524*	*1610*	*1629*	*1632*	*1646*	*1801*
**H1**	C	C	A	T	C	G	G	A	T	T	G	C	T	G	T	A	A	A	A	C	C	A	A	T	A	T	T	C	C	T	C	A
H2	•	•	•	•	•	A	•	•	•	•	•	•	•	•	C	•	•	-	•	•	•	•	•	C	•	•	•	•	T	•	•	•
H3	•	•	•	•	T	A	•	•	•	•	•	•	•	•	•	•	•	-	G	•	•	•	G	•	•	•	•	•	T	•	•	•
H4	•	•	•	C	•	A	•	•	•	•	A	T	C	•	•	•	-	-	G	•	T	•	•	•	•	•	•	T	T	•	T	•
H5	•	T	•	•	•	A	•	•	C	•	•	•	•	•	•	•	-	-	G	•	•	•	•	•	•	•	•	•	T	•	T	•
H6	•	T	•	•	•	A	•	•	C	•	•	•	•	A	•	•	-	-	G	•	•	•	•	•	•	•	•	•	T	•	T	•
H7	T	T	•	•	•	A	•	•	C	•	•	•	•	•	•	•	-	-	G	•	•	G	•	•	•	•	•	•	T	•	T	•
H8	T	T	•	•	•	A	•	•	•	C	•	•	•	•	•	•	-	-	G	•	•	G	•	•	•	•	•	•	T	•	T	•
**H9**	•	•	•	•	•	A	•	•	•	C	•	•	•	•	•	G	-	-	G	•	•	•	•	•	G	•	•	•	T	•	T	•
H10	•	•	•	•	•	A	•	•	•	C	•	•	•	•	•	•	-	-	G	•	•	•	•	•	G	C	•	•	T	•	T	•
**H11**	•	•	•	•	•	A	•	•	•	C	•	•	•	•	•	•	-	-	G	T	•	•	•	•	G	C	•	•	T	•	T	•
H12	•	•	•	•	•	A	A	•	•	C	•	•	•	•	•	•	-	-	G	T	•	•	•	•	G	C	•	•	T	•	T	•
**Z1**	•	•	•	•	•	A	•	•	•	C	•	•	•	•	•	•	-	-	G	•	•	•	•	•	G	C	•	•	T	C	T	•
**Z2**	•	•	•	•	•	A	•	•	•	C	•	•	•	•	•	•	-	-	G	•	•	•	•	•	G	C	•	•	T	C	T	T
**Z3**	•	•	•	•	•	•	•	•	•	•	A	•	•	•	•	•	•	•	•	•	•	•	•	•	•	•	•	•	•	•	•	•
**Z4**	•	•	G	•	•	A	•	•	•	C	•	•	•	•	•	G	-	-	G	•	•	•	•	•	G	•	•	•	T	•	T	•
**Z5**	•	•	•	•	•	A	•	G	•	C	•	•	•	•	•	G	-	-	G	•	•	•	•	•	G	•	•	•	T	•	T	•
**Z6**	•	•	•	•	•	A	•	•	•	C	•	•	•	•	•	•	-	-	G	T	•	•	•	•	G	C	C	•	T	•	T	•

AMOVA analysis, run as an eastern and western sub-population, resulted in an FST of 0.53 (p-value <0.001; Table 3). Gene diversity was calculated to be 0.7237 +/- 0.0112 for one Zambian population then 0.4712 +/- 0.0226 and 0.5041 +/- 0.0382 for the eastern and western sub-populations, respectively (S9 Appendix).

**Table 3:** AMOVA results with FST. Percent variation is given among populations (Va) and within groups (Vb). The significance of differentiation within and among populations was tested by 1,000 permutations.

*mtDNA*					
**Source of Variation**	**d.f.**	**Sum of Squares**	**Variance Components**	**Percentage of Variation**	**p-value**
Among Populations	1	232.299	1.237	53%	<0.001
Within Populations	385	419.076	1.089	47%	<0.001
Total	386	651.375	2.325		
**Fixation Index**	F_ST_:	0.532			
*STRs*					
**Source of Variation**	**d.f.**	**Sum of Squares**	**Variance Components**	**Percentage of Variation**	**p-value**
Among Populations	1	80.883	0.206	4%	<0.001
Within Populations	392	3720.861	4.801	96%	<0.001
Total	787	3801.745	5.007		
**Fixation Index**	F_ST_:	0.041			

**Fig 2:**
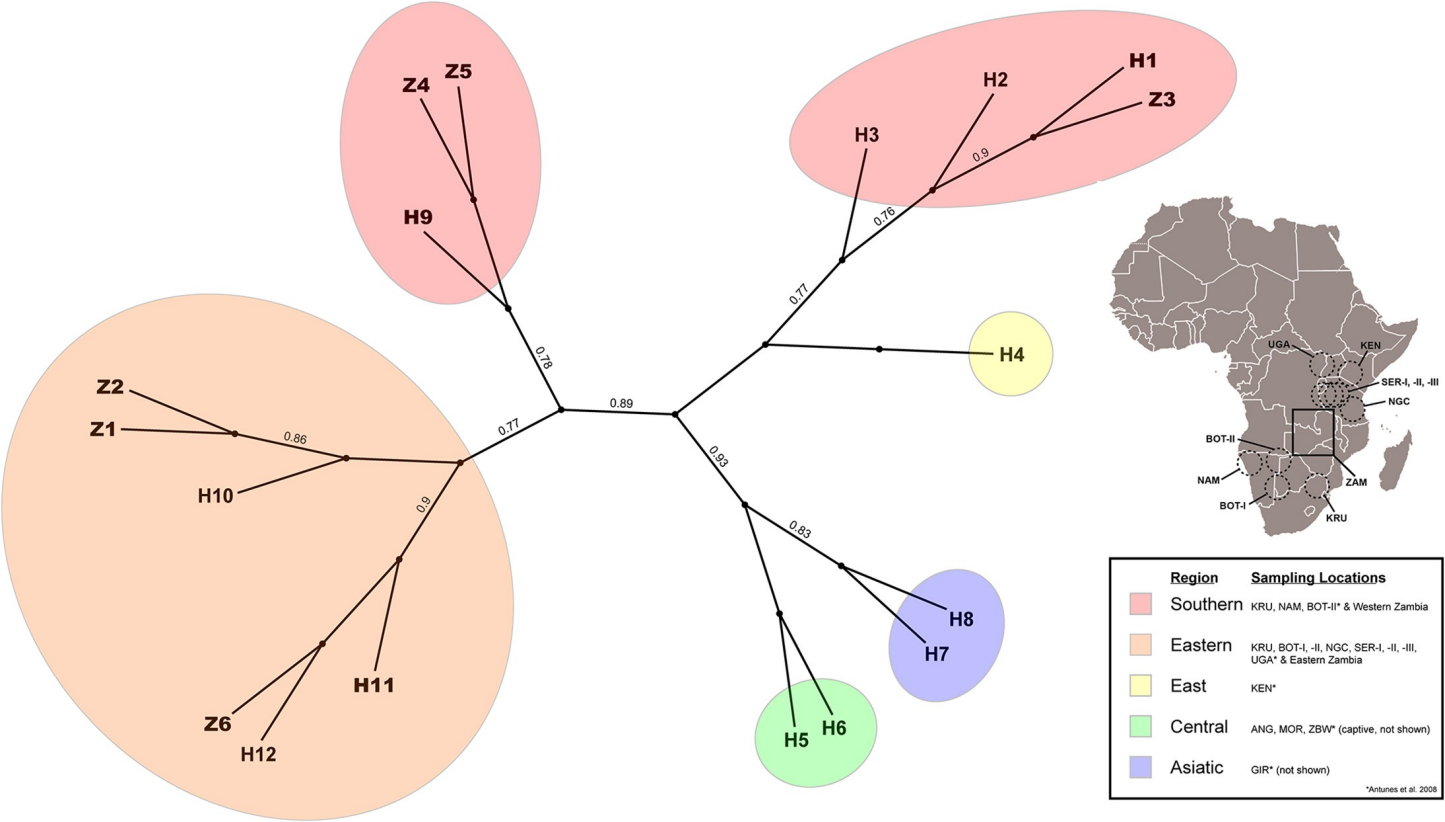
Radiated maximum likelihood tree with branch support. Clades are colored by region. Haplotypes in bold are found in Zambia. Map insert is from Curry et al. 2015 showing locations of lions sampled. Circles indicate geographic locations for populations determined by Antunes et al [15]. UGA (Uganda); KEN (Kenya); SER (Serengeti National Park, Tanzania); NGC (Ngorongoro Crater, Tanzania); KRU (Kruger National Park, South Africa); BOT-I (Southern Botswana and Kalahari, South Africa); BOT-II (Northern Botswana); NAM (Namibia); GIR (Gir Forest, India); ANG (Angola); ZBW (Zimbabwe); and MOR (Morocco). ZAM (Zambia) is denoted by a square.

All trees resulted in similar clustering. The unrooted maximum likelihood tree is shown (Fig 2). The general configuration of the phylogenetic tree and haplotype network did not change from Curry *et al*. [12], even with the addition of another novel haplotype (S3 Appendix). Haplotype Z6 appears as an additional branch to the H11 cluster, the second most predominant haplotype in the eastern sub-population.

### Microsatellite diversity

**Fig 3:**
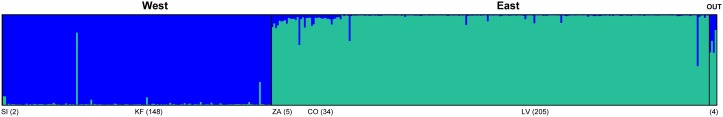
Results of STRUCTURE analysis based on 14 microsatellite loci of 398 Zambian lions. Population assignment results for ΔK = 2 showing a separation of the western and eastern lion populations in Zambia.

A total of 398 individuals were genotyped for 14 miniSTRs (S1 Appendix). Structure analysis revealed two sub-populations (ΔK = 2; Fig 3) in agreement with mtDNA structuring [12]. Luangwa Valley (LV), Corridor (CO) and Lower Zambezi (ZA) of the LVE make up the eastern sub-population and Kafue (KF) and Sioma Ngwezi (SI) of the GKE make up the western sub-population. Little admixture is evident between sub-populations. Two individuals are assigned to a sub-population contrary to the location they were sampled. Individual 2011000432 sampled in KF is assigned to the eastern sub-population (0.81) and 2011000878 from north of North Luangwa NP is almost equally assigned to the eastern (0.43) and the western (0.57) sub-populations.

Gene diversity, as established by expected heterozygosity (H_E_), is high (Table 4) locus by locus and as the mean across loci for the population (0.701) and within sub-populations (eastern = 0.682 and western = 0.692). Significant deviations for HWE are seen in 13 of 14 loci when the population is considered as a whole. Separated into sub-populations, the eastern sub-population deviates from HWE at 4 loci (Leo126, Leo224, Leo230, and Leo 391) while the western sub-population deviates from HWE at only 3 loci (Leo230, Leo247, Leo247). The only shared loci that deviates from HWE is Leo230. For most loci that deviate from HWE, a deficiency of heterozygotes is observed (Table 4).

**Table 4:** STR diversity indices.

Locus	N	A	AR	PA	PAR	H_O_	H_E_	p-value	Signif
**Leo006**	397	14	13.85			0.763	0.835	0.000	***
*East*	*243*	*12*	*10*.*69*	*3*	*2*.*71*	*0*.*774*	*0*.*773*	*0*.*054*	*ns*
*West*	*150*	*11*	*10*.*70*	*2*	*2*.*72*	*0*.*747*	*0*.*810*	*0*.*545*	*ns*
**Leo008**	398	8	7.96			0.729	0.762	0.003	***
*East*	*244*	*7*	*6*.*78*	*1*	*0*.*92*	*0*.*705*	*0*.*694*	*0*.*504*	*ns*
*West*	*150*	*7*	*6*.*84*	*1*	*0*.*98*	*0*.*760*	*0*.*738*	*0*.*640*	*ns*
**Leo031**	396	7	6.74			0.407	0.387	0.025	*
*East*	*244*	*7*	*6*.*06*	*4*	*3*.*06*	*0*.*377*	*0*.*364*	*0*.*072*	*ns*
*West*	*148*	*3*	*3*.*00*	*0*	*0*.*00*	*0*.*446*	*0*.*407*	*0*.*396*	*ns*
**Leo045**	396	8	7.72			0.356	0.410	0.005	**
*East*	*242*	*7*	*6*.*07*	*3*	*2*.*08*	*0*.*455*	*0*.*503*	*0*.*163*	*ns*
*West*	*150*	*5*	*4*.*96*	*1*	*0*.*98*	*0*.*193*	*0*.*226*	*0*.*059*	*ns*
**Leo077**	398	7	7.00			0.746	0.740	0.040	*
*East*	*244*	*7*	*7*.*00*	*1*	*1*.*00*	*0*.*758*	*0*.*748*	*0*.*102*	*ns*
*West*	*150*	*6*	*6*.*00*	*0*	*0*.*00*	*0*.*733*	*0*.*716*	*0*.*355*	*ns*
**Leo085**	398	9	8.86			0.668	0.654	0.192	ns
*East*	*244*	*6*	*5*.*87*	*2*	*1*.*87*	*0*.*635*	*0*.*618*	*0*.*093*	*ns*
*West*	*150*	*7*	*6*.*86*	*3*	*2*.*86*	*0*.*720*	*0*.*686*	*0*.*628*	*ns*
**Leo098**	398	8	7.86			0.661	0.702	0.013	*
*East*	*244*	*8*	*7*.*46*	*2*	*1*.*51*	*0*.*623*	*0*.*671*	*0*.*137*	*ns*
*West*	*150*	*6*	*6*.*00*	*0*	*0*.*05*	*0*.*727*	*0*.*706*	*0*.*088*	*ns*
**Leo126**	397	10	9.86			0.688	0.758	0.000	***
*East*	*243*	*10*	*9*.*43*	*3*	*1*.*85*	*0*.*658*	*0*.*744*	*0*.*000*	***
*West*	*150*	*8*	*7*.*58*	*0*	*0*.*00*	*0*.*740*	*0*.*757*	*0*.*335*	*Ns*
**Leo224**	398	9	8.98			0.668	0.711	0.000	***
*East*	*244*	*8*	*7*.*75*	*3*	*2*.*05*	*0*.*660*	*0*.*711*	*0*.*000*	***
*West*	*150*	*7*	*6*.*72*	*1*	*1*.*02*	*0*.*673*	*0*.*701*	*0*.*132*	*Ns*
**Leo230**	344	12	12.00			0.738	0.812	0.000	***
*East*	*211*	*10*	*9*.*55*	*2*	*1*.*55*	*0*.*701*	*0*.*795*	*0*.*000*	***
*West*	*129*	*10*	*10*.*00*	*2*	*2*.*00*	*0*.*798*	*0*.*825*	*0*.*000*	***
**Leo247**	398	9	8.96			0.759	0.806	0.000	***
*East*	*244*	*8*	*7*.*78*	*1*	*0*.*78*	*0*.*791*	*0*.*813*	*0*.*063*	*Ns*
*West*	*150*	*8*	*7*.*98*	*1*	*0*.*98*	*0*.*713*	*0*.*787*	*0*.*001*	***
**Leo281**	398	16	15.85			0.646	0.693	0.000	***
*East*	*244*	*15*	*13*.*83*	*2*	*1*.*72*	*0*.*635*	*0*.*634*	*0*.*289*	*Ns*
*West*	*150*	*14*	*13*.*57*	*1*	*1*.*46*	*0*.*667*	*0*.*758*	*0*.*000*	***
**Leo391**	397	9	8.98			0.695	0.740	0.040	*
*East*	*244*	*9*	*8*.*78*	*2*	*1*.*78*	*0*.*652*	*0*.*695*	*0*.*002*	**
*West*	*149*	*7*	*7*.*00*	*0*	*0*.*00*	*0*.*765*	*0*.*778*	*0*.*924*	*Ns*
**Leo506**	391	11	10.86			0.785	0.807	0.018	*
*East*	*242*	*9*	*8*.*10*	*2*	*2*.*40*	*0*.*777*	*0*.*793*	*0*.*082*	*ns*
*West*	*145*	*8*	*7*.*89*	*2*	*2*.*19*	*0*.*800*	*0*.*789*	*0*.*208*	*ns*
***Mean***	***393***	***9*.*79***	***9*.*68***			***0*.*665***	***0*.*701***	***0*.*024***	*
*East*	*241*	*8*.*79*	*8*.*22*	*2*.*21*	*1*.*81*	*0*.*657*	*0*.*682*	*0*.*112*	*ns*
*West*	*148*	*7*.*64*	*7*.*51*	*1*.*00*	*1*.*09*	*0*.*677*	*0*.*692*	*0*.*308*	*ns*

N, Sample Size; A, Number of Alleles; AR, Allelic Richness; PA, Number of Private Alleles; PAR, Private Allelic Richness; HO, Observed Heterozygosity; HE, Expected Heterozygosity; p-value for deviation from HWE: ns, not significant;

* p-value < 0.05;

** p-value < 0.01;

*** p-value < 0.001

Number of alleles (A) and allelic richness (AR) are high across loci (Table 4). The number of private alleles (PA) is high for both sub-populations. There are 213 individuals with 1–4 PA at 1–3 loci (Table 5). For the eastern sub-population, there are 31 PA spanning all 14 loci with 144 of 244 individuals having at least one PA. The western sub-population has 14 PA in nine of 14 loci and 69 out of 150 individuals have at least one PA.

**Table 5:** Number of individuals with private alleles (PA). N_#PA@#Loci_ is the number of private alleles at the given number of loci. Totals are given for the total number of individuals with any PA (N_wPA_), number of individuals homozygous for a PA (N_Hom4PA_), i.e. two of the same PA at the same locus for one or more loci, and number of individuals heterozygous for a PA (N_Hom4PA_), i.e. two different PA at the same locus for one or more loci.

	Eastern	Western	Total
**N**_**wPA**_	**144**	**69**	**213**
*N*_*wPA@1Locus*_	*86*	*46*	*132*
*N*_*wPA@2Loci*_	*44*	*23*	*67*
*N*_*wPA@3Loci*_	*14*	*0*	*14*
*N*_*wPA@>3Loci*_	*0*	*0*	*0*
**N**_**Hom4PA**_	**10**	**2**	**12**
*N*_*2PA@1Locus*_	*3*	*2*	*5*
*N*_*3PA@2Loci*_	*5*	*0*	*5*
*N*_*4PA@3Loci*_	*2*	*0*	*2*
**N**_**Het4PA**_	**6**	**1**	**7**
*N*_*2PA@1Locus*_	*3*	*1*	*4*
*N*_*3PA@2Loci*_	*2*	*0*	*2*
*N*_*4PA@3Loci*_	*1*	*0*	*1*

Weak but significant genetic structure is detected with an F_ST_ = 0.04 (p-value = 0.001; Table 3) attributing 4% of molecular variance among populations and 96% of molecular variance within populations. There is little evidence of inbreeding with an F_IS_ below zero (-0.034). The number of migrants per generation (Nm) was calculated to be 5.6 using two methods (GenAlEx and GenePop).

**Fig 4:**
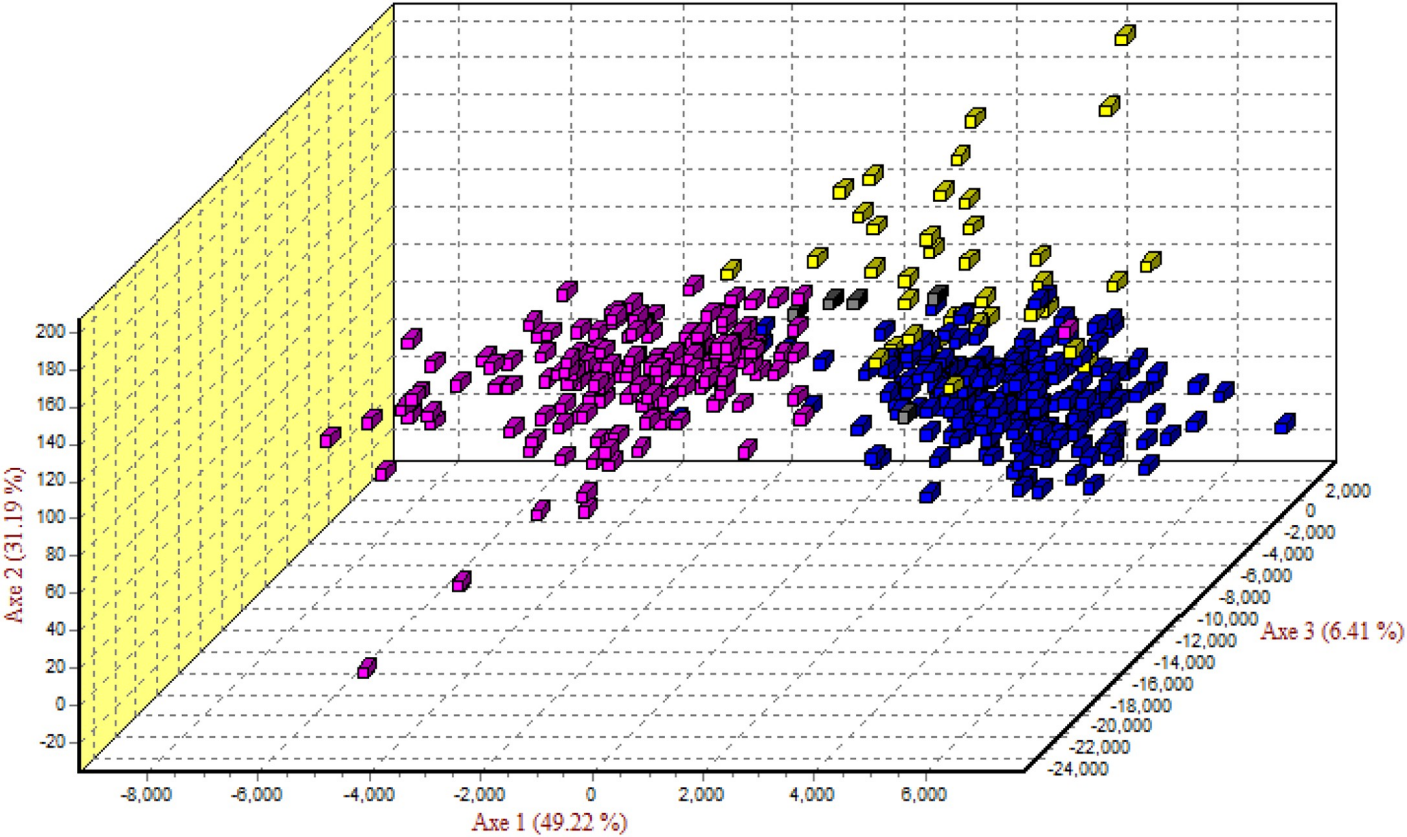
Factorial correspondence analysis (FCA) based on 14 microsatellite loci of 398 Zambian lions. Pink = Western Zambia (KF & SI), Blue = Luangwa Valley (LV), Yellow = Lower Zambezi NP and eastern corridor (ZA & CO), Grey = Outside PAs. Axe 1, 2, and 3 represent 86.82% of the genetic variation observed.

A Mantel test was done for males and females separately to account for possible dispersal differences between sexes due to their mating system [37–39]. Both males and females show a low but significant level of IBD (Male Rxy = 0.214, p-value = 0.01; Female Rxy = 0.269, p-value = 0.01; S5 Appendix). Nei’s distances are higher between western and eastern regions (S7 Appendix). Factorial Correspondence Analysis (FCA; Fig 4) and Principle Coordinate Analysis (PCoA; S6 Appendix) reveal that LV and KF cluster separately with CO and ZA as intermediaries, overlapping with both LV and KF.

**Fig 5:**
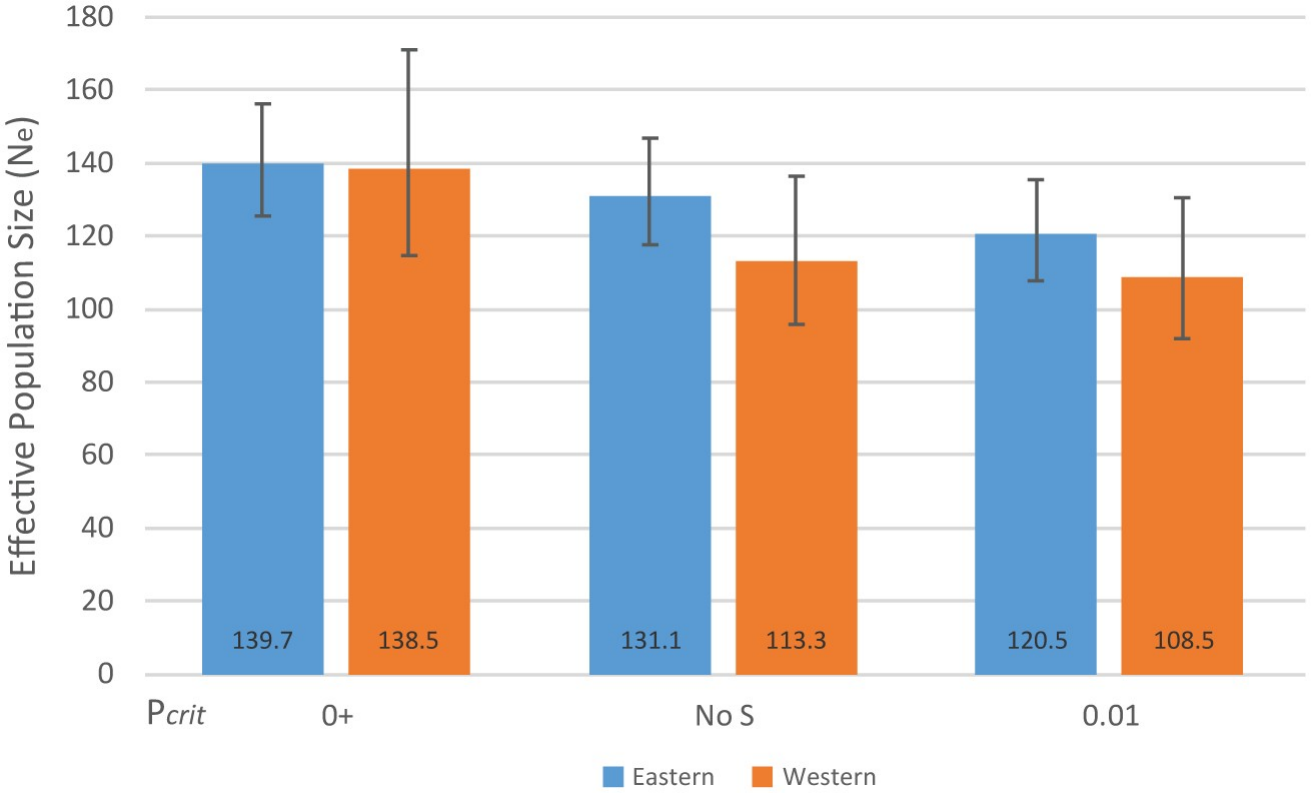
Effective population size (N_e_) calculations with upper 95% confidence intervals. Estimates of N_e_ with P_*crit*_ set to frequencies of 0.00 (0+), 0.01, and to exclude only singleton alleles (No S).

Effective population size (N_e_) calculations with upper 95% confidence intervals are shown in Fig 5 for the eastern and western sub-populations.

## Discussion

Increasing the sample size did not change the gene diversity of lions within the western sub-population of Zambia, nor did it change gene diversity at the country-wide scale [12]. Gene diversity of the eastern sub-population was lowered slightly, but still remains high (S9 Appendix). The addition of microsatellite analysis supports earlier findings that Zambia’s lion population is highly diverse (Table 4). Furthermore, with an F_IS_ value close to zero, Zambian lions do not show any evidence of inbreeding.

The addition of another novel haplotype (Z6) did not change the configuration of the phylogenetic tree or haplotype network. Haplotype Z6 is one SNP from H11, clustering with H11 and H12 within the East/Southern Africa cluster. H11 is the most wide-spread haplotype, found in individuals as far north as Uganda and south as Kruger NP [15]. Most Zambian haplotypes cluster together within two branches of the East/Southern Africa cluster. Haplotype Z3, found only in Kafue NP, is in a different cluster with other southern haplotypes. This clustering suggests connectivity of the western sub-population southwest while the eastern sub-population has connectivity both north and south but remains to the east.

Mitochondrial analysis shows minimal gene flow between populations (F_ST_ = 0.53) while microsatellite analysis suggests greater gene flow (F_ST_ = 0.04). F_ST_ for microsatellites is the measure of the heterozygote deficit relative to its expectation under HWE [40] while F_ST_ for mtDNA is a function of the number of mutations between molecular haplotypes as measured by haploid diversity [18]. These differences in concept could also be a reason for the vast differences between the two F_ST_ values. However, this pattern could also be a result of the mating system where males disperse across farther distances than females [37]. Males are more likely to disperse, passing along their nDNA but unable to pass on mtDNA genes.

Pairwise differences (S7 Appendix) for nDNA and mtDNA also show evidence of the mating system. For nDNA distances are lower between ZA in the east and KF in the west, indicating this is likely the region genetic movement between the eastern and western sub-populations occur. Further evidence of this movement is shown in FCA analysis (Fig 4).

Migration is the transfer of genetic variation from one population to another [41]. Mutation cannot be disentangled from differences introduced by migration so F_ST_ can underestimate differentiation in a highly structured population [40], particularly when other metrics support strong structure. Migration is evident within Zambia (Nm = 5.6), therefore, F_ST_ calculated for microsatellites, could be an underestimation based on high migration coupled with the distinct structure shown in other analyses (Figs 3 and 4). However, as migration increases, the proportion of private alleles will decrease. If gene flow is low, there are more private alleles, and if gene flow is high, private alleles are more rare [41]. Zambia has a high number of private alleles with 31 private alleles in the eastern sub-population and 13 in the western sub-population found in 213 individuals. A majority of these alleles are in low frequency, however, 13 appear in frequencies greater than 1% of the sampled population.

Structure analysis shows two distinct sub-populations with admixture present in only a few individuals (Fig 3). 2011000878, sampled in a GMA north of North Luangwa NP, is the most admixed individual, assigned almost equally to each sub-population, implying it is the offspring of a resident and migrant mating. A possible migrant, 2011000432, was sampled in the western sub-population but is assigned to the eastern sub-population. This same individual was previously flagged as a possible migrant based on its mtDNA haplotype [12].

Further support for substructure is the presence of a Wahlund effect. This is when the subdivision of genetically distinct demes causes a deviation from HWE at the population level resulting in the appearance of a deficit of heterozygotes [42–45]. Across Zambia, mean H_O_ versus H_E_ shows a heterozygote deficiency at the population level, however, when separated into sub-populations, mean values no longer deviate from HWE (Table 4). This same pattern is present across loci with a deviation from HWE at the population level and being in HWE at sub-population level. Leo230 is the only locus that remains out of HWE at the sub-population level. This may be a result of issues with the locus, as this was the only locus that exhibited problems with amplification.

With evidence of clusters, substructure, and Wahlund effects, it is suggested to remove migrants from the population before estimating N_e_ [46]. Therefore, migrants and individuals found outside PAs were removed before calculating N_e_. The LD method was used because it is robust and mostly unbiased at a sub-population level [47]. In simulations of single-sample N_e_ estimators, the LD method performed best producing estimations of N_e_ closest to the true value of N_e_ [47–49]. Heterozygote excess and molecular co-ancestry methods often have poor precision in comparison [46,49].

The population size of lions in Zambia during the sampling period was estimated to be as low as 700 lions with 250–500 in Kafue NP, 400–750 in the Luangwa Valley, and <50 in the corridor and Lower Zambezi NP [50]. N_e_ for the eastern and western sub-populations is calculated to be between 100–200, a lower value than what would be expected for a large and diverse lion population [51]. Lion prides typically have multiple related females mating with 1–7 males that originated from a different pride or prides [37]. This type of polygynous mating system can lower the value of N_e_ depending on the number of males breeding within each pride [51].

When loci are physically unlinked, LD is caused by drift, migration, or selection. Assuming neutral loci in an isolated population with random mating, LD would be a result of drift alone and can be used to calculate N_e_ [52]. However, LD calculated from a sample from a sub-population can lead to an underestimation of local N_e_ when the migration rate is low [47,49]. The number of migrants between the eastern and western sub-populations is calculated to be less than six individuals per generation. This is a migration rate well below 5–10% [47]. Therefore, this could be an underestimation of N_e_ (Fig 5) as a result of migration between sub-populations.

Overall, Zambia has a genetically diverse population of lions, although effective population size appears to be lower than expected. Previously thought to be isolated via anthropogenic and geographic barriers, the eastern and western sub-populations do exhibit isolation-by-distance, with a low level of migrants per generation. This migration may cause an underestimation of effective population size but is maintaining and introducing diversity across Zambia.

Translocation is a well‐practiced technique to prevent inbreeding [53]. Zambia does not appear to be in need of using translocation as a management strategy. While maintaining genetic diversity throughout the entire population should be considered, the high number of private alleles present within each sub-population and the level of population substructure found suggests there should be a more narrowed focus to prevent the loss of genetic diversity within sub-populations. Maintenance of diversity across Zambia will still occur through gene flow of lions between sub-populations, as it has been occurring already without intervention. This is assuming that future connectivity between sub-populations stays the same, or improves, rather than decreasing.

Range-wide studies have proposed lions are mostly structured by region due to restricted widespread movement of lions across the landscape [15,54,55]. Findings in this study agree, though movement outside protected areas is occurring. To further augment gene flow, historic or present-day corridors would need to be created and protected to help ensure continued natural dispersal of lions between the eastern and western sub-populations.

## Supporting information

S1 Appendix Sample information. (XLSX)

S2 Appendix Protocols and procedures. (XLSX)

S3 Appendix Additional phylogenetic analyses. (PDF)

S4 Appendix Structure Harvester graphical output. (PDF)

S5 Appendix Mantel tests. (PDF)

S6 Appendix Principle coordinate analysis. (PDF)

S7 Appendix Pairwise differences. (PDF)

S8 Appendix Private allele frequencies. (PDF)

S9 Appendix Comparative molecular diversity indices and nucleotide composition. (PDF)
