# Supplementary material for: Genetic analysis of African lions (Panthera leo) in Zambia support movement across anthropogenic and geographical barriers
Source: PLoS One. 2019 May 31;14(5):e0217179. doi: 10.1371/journal.pone.0217179 (PMC6544237; doi:10.1371/journal.pone.0217179)
Supplement: S3 Appendix — (PDF) [file pone.0217179.s003.pdf]

S3: Additional Phylogenetic Analyses

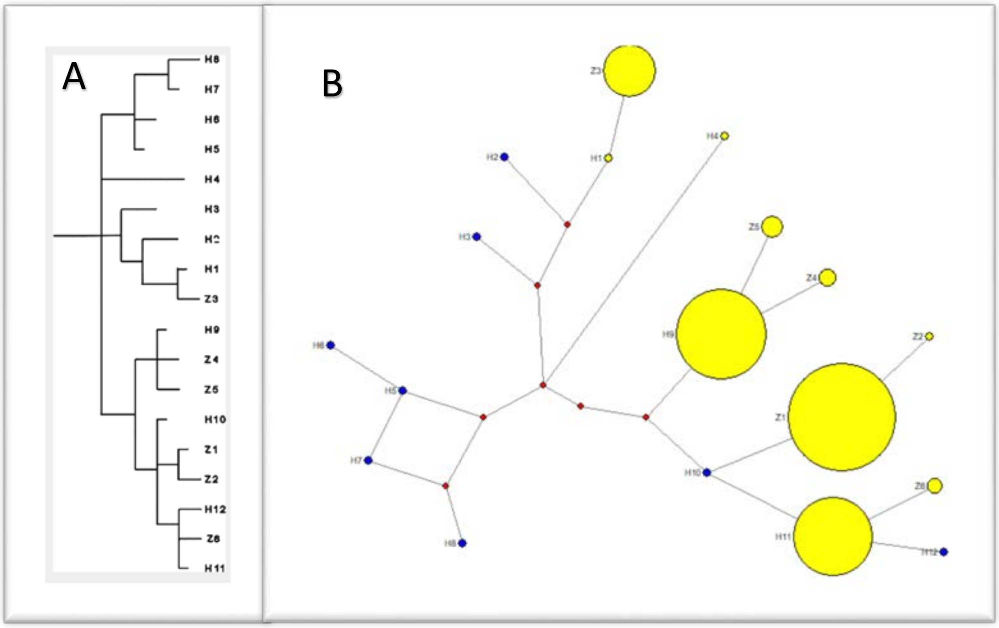

(A) Bayesian analysis. H1-H12 are haplotypes that were described by Antunes et al. 2008 and Z1-Z5 are novel haplotypes so far only found within Zambia.

(B) Median-joining network of 12S-16S haplotypes. Yellow indicates haplotypes found Zambia. Circle sizes of haplotypes found in Zambia are proportional to haplotype frequency. Red circles indicate median vectors. Blue circles indicate haplotypes not found in Zambia. H1-H12 are haplotypes which were described by Antunes et al. 2008 and Z1-Z5 are novel haplotypes so far only found within Zambia.

Antunes A, Troyer JL, Roelke ME, Pecon-Slattery J, Packer C, Winterbach C, et al. The evolutionary dynamics of the lion Panthera leo revealed by host and viral population genomics. PLoS Genet. 2008;4: e1000251.
