# Supplementary material for: Genetic analysis of African lions (Panthera leo) in Zambia support movement across anthropogenic and geographical barriers
Source: PLoS One. 2019 May 31;14(5):e0217179. doi: 10.1371/journal.pone.0217179 (PMC6544237; doi:10.1371/journal.pone.0217179)
Supplement: S4 Appendix — (PDF) [file pone.0217179.s004.pdf]

**S4:** Structure Harvester Graphical Output from Evanno Method*

L(K)

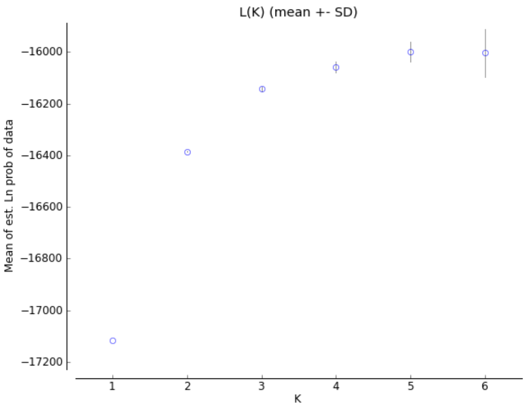


**L'(K)**

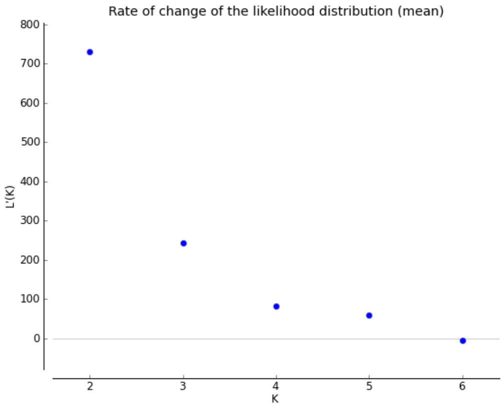


**|L''(K)|**

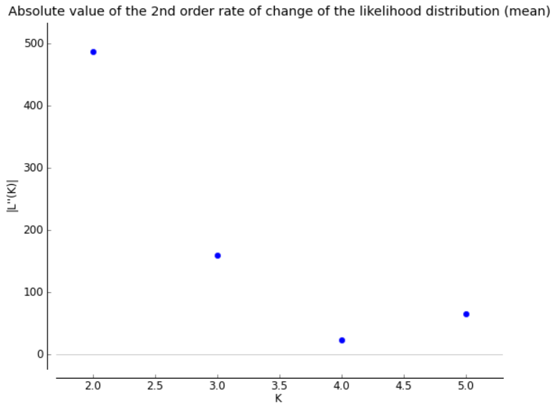


**ΔK**

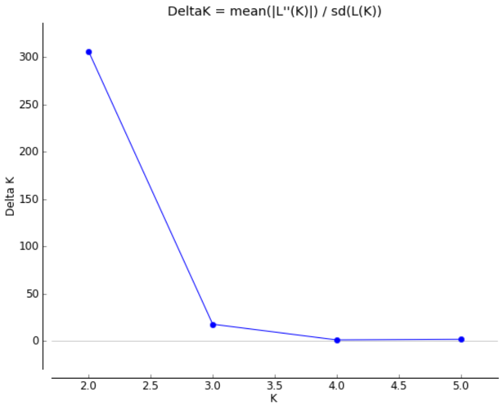


*Earl DA, vonHoldt BM. STRUCTURE HARVESTER: A website and program for visualizing STRUCTURE output and implementing the Evanno method. Conserv Genet Resour. 2012;4: 359–361. doi:10.1007/s12686-011-9548-7
