# Supplementary material for: Genetic analysis of African lions (Panthera leo) in Zambia support movement across anthropogenic and geographical barriers
Source: PLoS One. 2019 May 31;14(5):e0217179. doi: 10.1371/journal.pone.0217179 (PMC6544237; doi:10.1371/journal.pone.0217179)
Supplement: S5 Appendix — (PDF) [file pone.0217179.s005.pdf]

S5: Genetic distance vs geographic distance Mantel Tests for Matrix Correspondence and Spatial Autocorrelation Analysis calculated in GenAlEx.

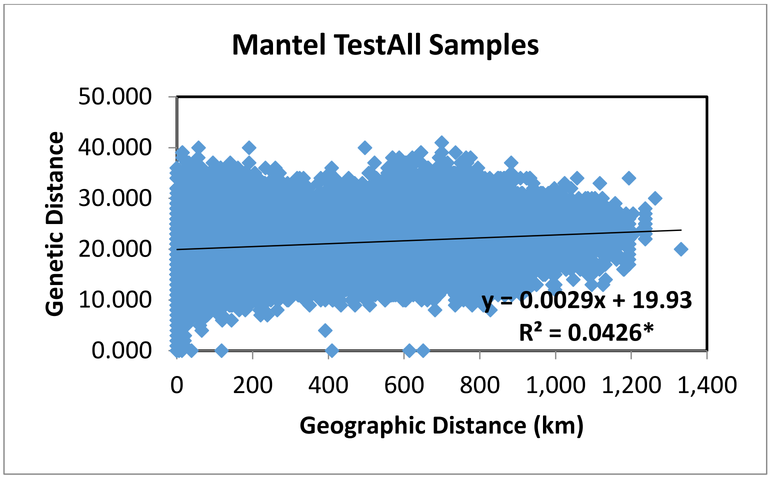

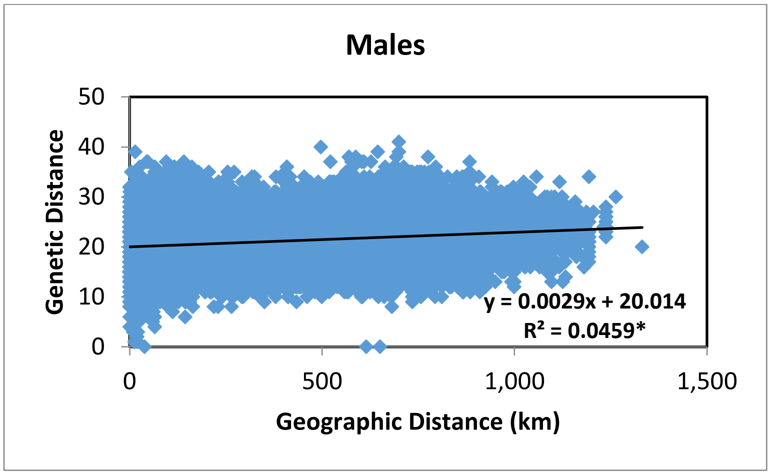

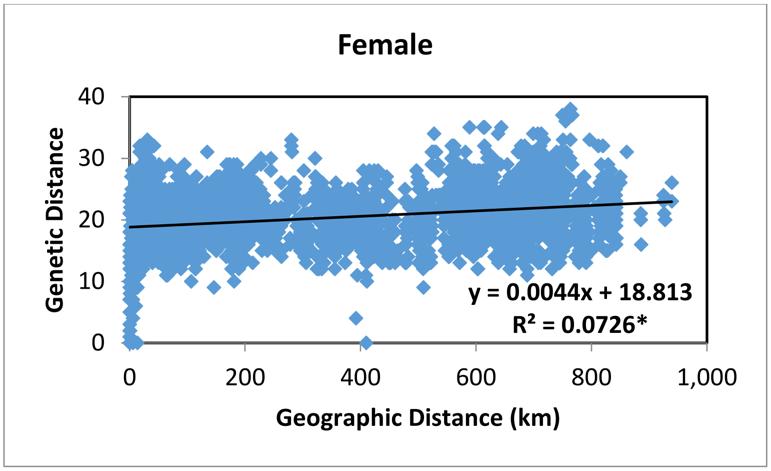

*p-value < 0.01
