# Supplementary figures and images for: Genetic analysis of African lions (Panthera leo) in Zambia support movement across anthropogenic and geographical barriers
Source: PLoS One. 2019 May 31;14(5):e0217179. doi: 10.1371/journal.pone.0217179 (PMC6544237; doi:10.1371/journal.pone.0217179)

**S6:** Principle Coordinate Analysis (PCoA) calculated in GenAEx from genetic distance matrix.

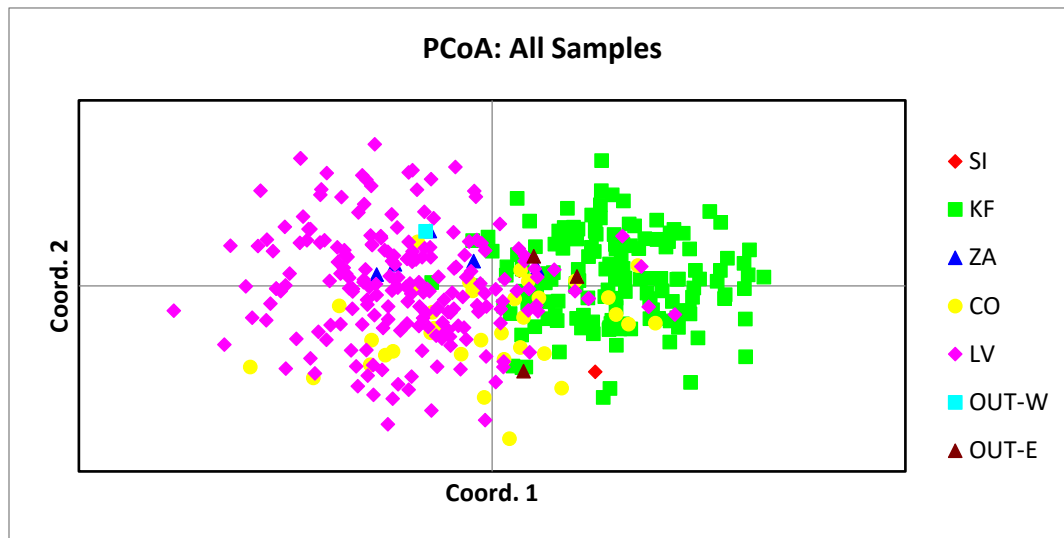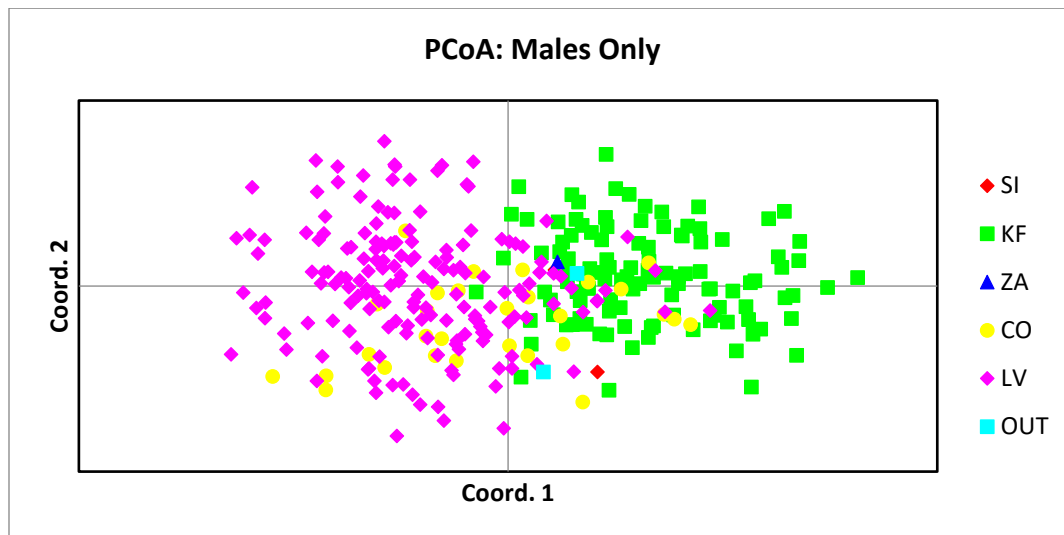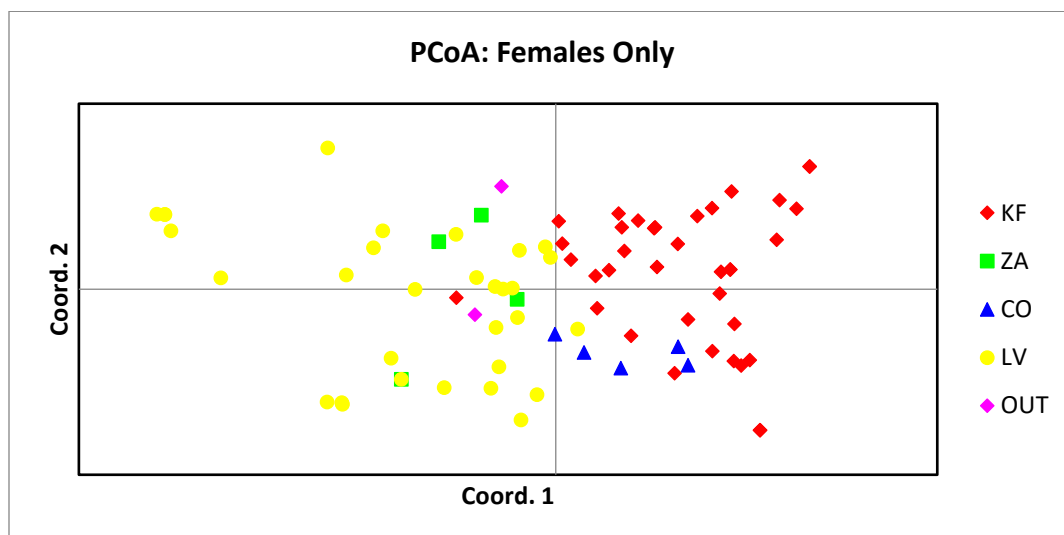

Supplement: S6 Appendix — (PDF) [file pone.0217179.s006.pdf]
