# Supplementary material for: Genetic analysis of African lions (Panthera leo) in Zambia support movement across anthropogenic and geographical barriers
Source: PLoS One. 2019 May 31;14(5):e0217179. doi: 10.1371/journal.pone.0217179 (PMC6544237; doi:10.1371/journal.pone.0217179)

S7 Pairwise Differences

```
Above diagonal     : Average number of pairwise differences between populations (PiXY)
Diagonal elements : Average number of pairwise differences within population (PiX)
Below diagonal     : Corrected average pairwise difference (PiXY-(PiX+PiY)/2)
```

**STRs**	LV	CO	ZA	KF	SI
LV	9.30231	9.3792	9.5061	9.83053	9.05183
CO	0.27787	8.90035	9.43529	9.6252	8.95956
ZA	-0.05617	0.07401	9.82222	9.83446	9.425
KF	0.47297	0.46862	0.21695	9.4128	8.90287
SI	-0.01599	0.09272	0.09722	-0.2202	8.83333

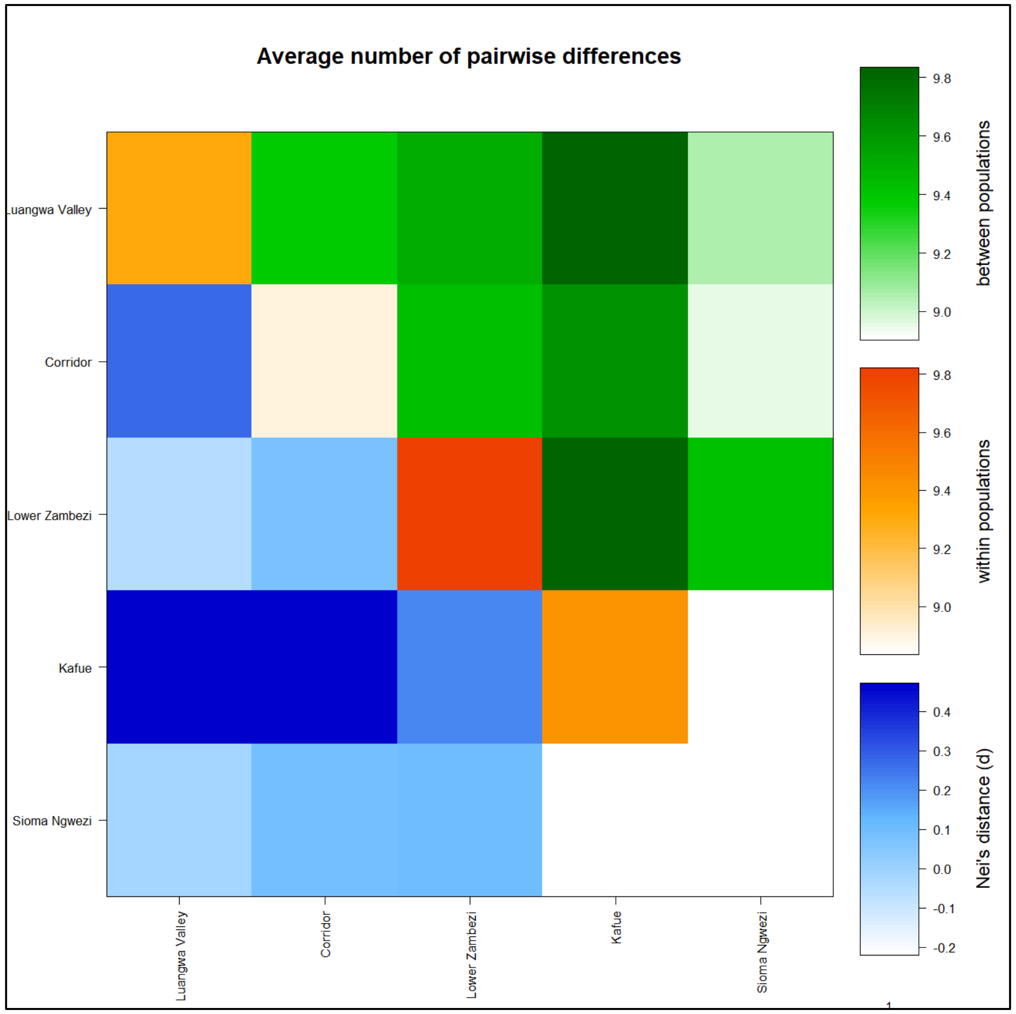


```
Above diagonal     : Average number of pairwise differences between populations (PiXY)
Diagonal elements : Average number of pairwise differences within population (PiX)
Below diagonal     : Corrected average pairwise difference (PiXY-(PiX+PiY)/2)
```

**mtDNA**	LV	CO	ZA	KF	SI
LV	0.88234	1.1175	1.30155	4.96055	2.4715
CO	0.14085	1.07097	1.2	5.00208	2.31183
ZA	0.06038	-0.13548	1.6	5.17032	2.46667
KF	2.53396	2.48118	2.3849	3.97084	3.38495
SI	1.03033	0.77634	0.66667	0.39953	2

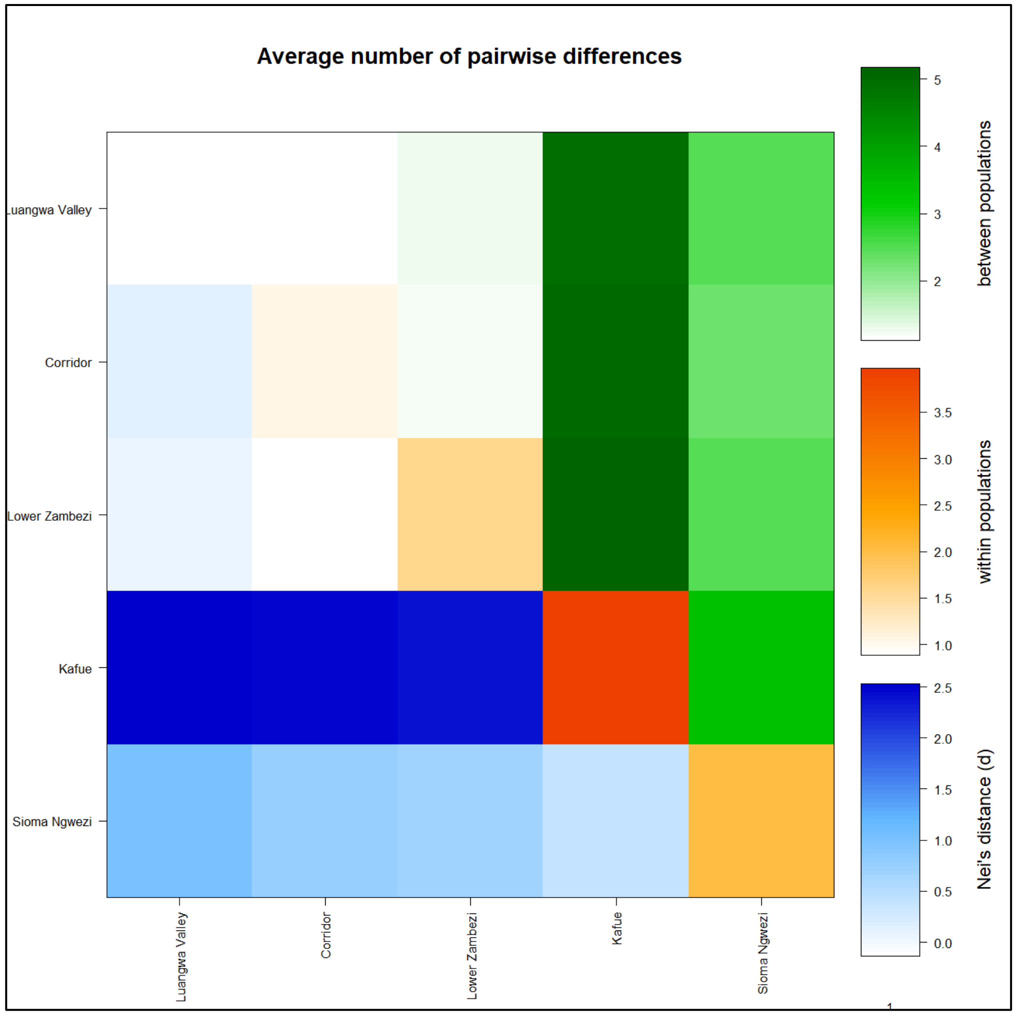

Supplement: S7 Appendix — (PDF) [file pone.0217179.s007.pdf]
