# Supplementary material for: Genetic analysis of African lions (Panthera leo) in Zambia support movement across anthropogenic and geographical barriers
Source: PLoS One. 2019 May 31;14(5):e0217179. doi: 10.1371/journal.pone.0217179 (PMC6544237; doi:10.1371/journal.pone.0217179)
Supplement: S8 Appendix — (PDF) [file pone.0217179.s008.pdf]

S8: Private Allele Frequencies

	Pop	Locus	Allele	$Freq_{GenAlEx}$	$N_{ALLELES}$	$N_{TotAlleles}$	Freq	N_{IND}	N_{Hom4PA}
1	Luangwa Valley	Leo006	128	0.023	11	802	0.014	11	0
2	Luangwa Valley	Leo006	132	0.008	4	802	0.005	4	0
3	Luangwa Valley	Leo006	136	0.002	1	802	0.001	1	0
4	Corridor	Leo008	133	0.004	2	804	0.002	2	0
5	**East**	Leo031	186	0.023	11	800	0.014	11	0
6	Luangwa Valley	Leo031	190	0.002	1	800	0.001	1	0
7	**East**	Leo031	194	0.045	22	800	0.028	21	**1**
8	Luangwa Valley	Leo031	200	0.002	1	800	0.001	1	0
9	Luangwa Valley	Leo045	72	0.002	1	800	0.001	1	0
10	Luangwa Valley	Leo045	96	0.021	10	800	0.013	10	0
11	Luangwa Valley	Leo045	108	0.002	1	800	0.001	1	0
12	**East**	Leo077	112	0.098	48	804	0.060	47	**1**
13	**East**	Leo085	70	0.006	3	804	0.004	3	0
14	Corridor	Leo085	92	0.010	5	804	0.006	4	**1**
15	Corridor	Leo098	96	0.002	1	804	0.001	1	0
16	**East**	Leo098	100	0.010	5	804	0.006	5	0
17	Luangwa Valley	Leo126	103	0.006	3	802	0.004	3	0
18	Luangwa Valley	Leo126	123	0.002	1	802	0.001	1	0
19	**East**	Leo126	143	0.029	14	802	0.017	13	**1**
20	Luangwa Valley	Leo224	84	0.004	2	804	0.002	1	**1**
21	Luangwa Valley	Leo224	88	0.018	9	804	0.011	9	0
22	**East**	Leo224	96	0.066	32	804	0.040	28	**4**
23	Corridor	Leo230	94	0.007	3	694	0.004	3	0
24	Luangwa Valley	Leo230	96	0.002	1	694	0.001	1	0
25	Luangwa Valley	Leo247	130	0.004	2	804	0.002	2	0
26	Corridor	Leo281	225	0.002	1	804	0.001	1	0
27	**East**	Leo281	253	0.004	2	804	0.002	2	0
28	Corridor	Leo391	166	0.004	2	802	0.002	2	0
29	Luangwa Valley	Leo391	170	0.043	21	802	0.026	20	**1**
30	Luangwa Valley	Leo506	189	0.002	1	790	0.001	1	0
31	**East**	Leo506	199	0.023	11	790	0.014	11	0
1	Kafue	Leo006	96	0.093	28	802	0.035	26	**2**
2	Kafue	Leo006	110	0.040	12	802	0.015	12	0
3	Kafue	Leo008	123	0.007	2	804	0.002	2	0
4	Kafue	Leo045	104	0.007	2	800	0.003	2	0
5	Kafue	Leo085	72	0.003	1	804	0.001	1	0
6	Kafue	Leo085	86	0.030	9	804	0.011	9	0
7	**West**	Leo085	96	0.010	3	804	0.004	3	0
8	Kafue	Leo224	90	0.010	3	804	0.004	3	0
9	Kafue	Leo230	74	0.035	9	694	0.013	9	0
10	Kafue	Leo230	86	0.008	2	694	0.003	2	0
11	Kafue	Leo247	136	0.007	2	804	0.002	2	0
12	**West**	Leo281	211	0.033	10	804	0.012	10	0
13	**West**	Leo506	187	0.017	5	790	0.006	5	0
14	**West**	Leo506	197	0.069	20	790	0.025	20	0

	N_{PA}			$N_{INDwAllPA@Locus}$		
	**East**	**West**	**Total**	**East**	**West**	**Total**
Leo006	3	2	5	1	3	4
Leo008	1	1	2	0	0	0
Leo031	4	0	4	2	0	2
Leo045	3	1	4	0	0	0
Leo077	1	0	1	1	0	1
Leo085	2	3	5	1	0	1
Leo098	2	0	2	0	0	0
Leo126	3	0	3	2	0	2
Leo224	3	1	4	8	0	8
Leo230	2	2	4	0	0	0
Leo247	1	1	2	0	0	0
Leo281	2	1	3	0	0	0
Leo391	2	0	2	1	0	1
Leo506	2	2	4	0	0	0
Totals	***31***	***14***	***45***	***16***	***3***	***19***
