# Supplementary material for: Genetic analysis of African lions (Panthera leo) in Zambia support movement across anthropogenic and geographical barriers
Source: PLoS One. 2019 May 31;14(5):e0217179. doi: 10.1371/journal.pone.0217179 (PMC6544237; doi:10.1371/journal.pone.0217179)
Supplement: S9 Appendix — (PDF) [file pone.0217179.s009.pdf]

S9. Comparative molecular diversity indices and nucleotide composition.

	Curry *et al.* 2015	This study
**Nucleotide Sites**	1882	1882
**# Haplotypes**	8	9
**Polymorphic Sites**	16	17
**Transitions**	13	14
**Transversions**	1	1
**Indels**	2	2
**Composition**		
**C**	22.11	22.12
**T**	22.67	22.66
**A**	36.64	36.65
**G**	18.58	18.57
**Diversity**		
**Overall**	0.7319 +/- 0.0174	0.7237 +/- 0.0112
**Eastern**	0.5057 +/- 0.0575	0.4712 +/- 0.0226
**Western**	0.5014 +/- 0.0336	0.5041 +/- 0.0382
**F_{ST}**	0.47	0.53

Curry, C. J., White, P. A., & Derr, J. N. (2015). Mitochondrial Haplotype Diversity in Zambian Lions: Bridging a Gap in the Biogeography of an Iconic Species. *Plos One*, *10*(12), e0143827. http://doi.org/10.1371/journal.pone.0143827
